# Tension and Compression Properties of 3D-Printed Composites: Print Orientation and Strain Rate Effects

**DOI:** 10.3390/polym15071708

**Published:** 2023-03-29

**Authors:** Tom Fisher, José Humberto S. Almeida Jr, Brian G. Falzon, Zafer Kazancı

**Affiliations:** 1 Advanced Composites Research Group, School of Mechanical and Aerospace Engineering, Queen’s University Belfast, Belfast BT9 5AH, UK; 2 School of Engineering, RMIT University, Melbourne, VIC 3000, Australia

**Keywords:** additive manufacturing, polymer composites, discontinuous fibre reinforcement, material characterisation, strain rate

## Abstract

This study examines the impact of three factors on the tensile and compressive behaviour of 3D-printed parts: (1) the addition of short carbon fibres to the nylon filament used for 3D printing, (2) the infill pattern, and (3) the speed at which the materials are strained during testing. The results show that adding carbon fibres to the nylon filament reduces variability between tests and emphasises the effect of print orientation. When the infill pattern is aligned with the direction of loading, the tensile strength of all samples increases, with the largest increase of 100% observed in the carbon fibre-reinforced samples, compared to a 37% increase in the strength of nylon samples. The carbon fibre-reinforced samples are also highly dependent on strain rate, with a 60% increase in tensile strength observed at a faster testing speed of 300 mm/min (9 min${}^{-1}$) compared to 5 mm/min (0.15 min${}^{-1}$). Nylon samples show a decrease of approximately 10% in tensile strength at the same increased speed. The compressive strength of the composite samples increases by up to 130% when the print path is parallel to the loading direction. Increases of up to 50% are observed in the compressive modulus of the composite samples at a test speed of 255 mm/min (9 min${}^{-1}$) compared to 1.3 mm/min (0.05 min${}^{-1}$). Similar trends are not seen in pure nylon samples. This study is the first to report on the variation of Poisson’s ratio of short carbon fibre-reinforced 3D-printed parts. The results show increases of up to 34% and 76% in the tensile and compressive Poisson’s ratios, respectively, when printing parameters are altered. The findings from this research will contribute to the design and numerical modelling of 3D-printed composites.

## 1. Introduction

Additive manufacturing (AM), or 3D printing, is rapidly advancing, enabling the production of more intricate parts. This advancement has expanded its use as a production technique for end-use products in various industries, including biomedical [1,2,3,4], automotive [5,6], sport [7,8,9] and consumer products [10,11,12]. With the emergence of new techniques, it is now possible to print high-performance materials, such as composites. Additive manufacturing encompasses a wide range of techniques, such as stereolithography (SLA), selective laser sintering (SLS) and fused filament fabrication (FFF) [13]. Both SLA and FFF techniques have the capability of producing composite parts either by the incorporation of continuous fibres or the addition of short fibres to the polymer matrix [14]. An advantage of FFF for short fibre-reinforced polymers compared to SLA is the alignment of the fibres, which takes place due to the extrusion process [15]. This may allow for the easier tailoring of mechanical properties within a part based on the infill direction [16].

In order to take full advantage of these 3D-printing methods, understanding the properties and the dependence on process parameters is important to optimise performance [17,18,19]. Before additively manufactured composites can become widespread in critical applications, such as defence and aerospace, the influence of certain loading conditions and strain rate is crucial to be understood.

Many researchers have investigated the mechanical behaviour of continuous fibre-reinforced 3D-printed specimens [20,21,22,23,24,25], with the majority focusing on tensile behaviour. Li et al. [24] investigated hybrid (carbon and Kevlar) continuous fibre-reinforced polymer. They found that an infill ±45° to the loading direction resulted in the lowest modulus and strength, followed by a 90° infill, with an infill parallel to the loading direction yielding the strongest parts with the highest modulus. Li et al. [25] investigated both the tension and compression behaviour of continuous fibre-reinforced polyamide. They reported very similar stress–strain curves for quasi-static and dynamic tensile tests but noticed a difference in failure surface, with a uniform failure in the quasi-static samples and deeper, more severe cracks in the dynamic failure surface caused by large bundles of fibres being pulled out. In compression, both the height and fibre arrangement of the specimens had a large effect on their behaviour. Although digital image correlation (DIC) was used in this study, there was no investigation into the Poisson’s ratio of the reinforced specimens.

Fewer researchers have investigated the influence of short-fibre orientation effects on the mechanical performance of 3D-printed parts. Casamento et al. [26] found that the inclusion of short carbon fibres was superior at increasing mechanical performance compared to carbon nanotubes. Peng et al. [27] investigated the tensile properties of short carbon fibre-reinforced polyamide-6 printed with an infill direction at 90°, ±45° and 0° to the loading direction, finding an increase in Young’s modulus and tensile strength with reducing the infill angle. Bakis et al. [28] and Abderrafai et al. [29] found similar results when comparing transverse and longitudinal printed specimens. Yasa et al. [30] compared the tensile properties of short carbon fibre-reinforced nylon printed in two orientations on the build plate. They found an increase in the Young’s modulus and yield strength for the samples printed on their edge compared to flat on the build plate, at a cost of reducing the elongation to failure. These researchers focused on tensile testing and neglected to consider compression, strain rate or the effect of these parameters on the resulting Poisson’s ratio of the material.

In this paper, the effect of short carbon fibre reinforcement, infill orientation and strain rate on the tensile and compressive behaviour of 3D-printed specimens is explored. The impact of these parameters on the mechanical properties, such as moduli, yield stress and elongation, are compared. The Poisson’s ratio of additively manufactured material has not been investigated in detail in the literature, and as such, is explored in this work. This study aims to deliver a deeper understanding of the effect of these parameters on the mechanical properties of 3D-printed composites, which will contribute to improving the design of additively manufactured parts as well as their numerical modelling, and could be used to vary the mechanical properties of a part throughout its structure.

## 2. Materials and Methods

In this study, two materials, produced by Markforged, were analysed: “Nylon White” an engineering-grade nylon released in 2019 and “Onyx”, a nylon reinforced with 14 wt% [31] short carbon fibres approximately 130 μm in length [32]. Test samples were manufactured using the Markforged Mark Two 3D printer, which uses a fused filament fabrication (FFF) technique. Uniaxial tensile and compression tests were carried out at a range of strain rates using a universal Zwick Z100 Testing Machine. The resulting strain fields in the samples were captured using an LA Vision Digital Image Correlation (DIC) system.

### 2.1. Additive Manufacture of Test Specimens

Sample designs were exported as stereolithography (STL) files and uploaded into Markforged’s slicing software, *Eiger* [33]. All pathing was carried out in *Eiger* and the parts were printed on the Markforged Mark Two printer with a layer height of 0.2 mm. Adjusting the pathing parameters within *Eiger* allows for the infill to be varied.

**Figure 1:**
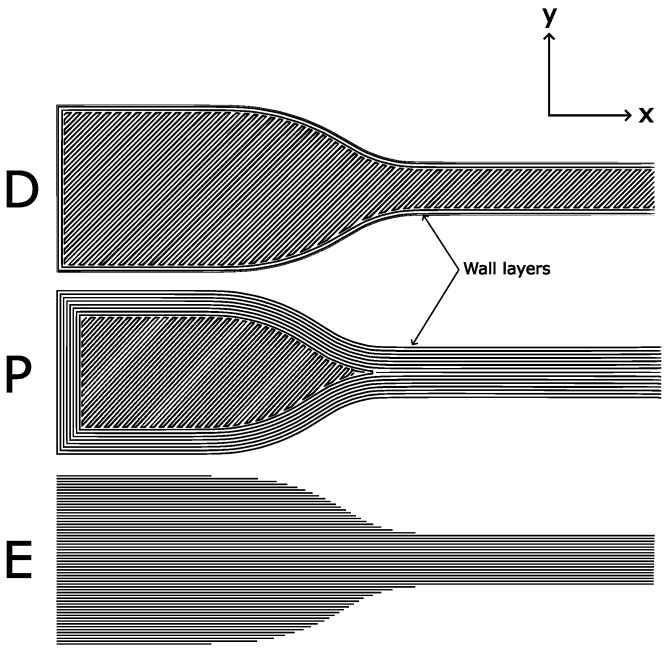
Infill of tensile specimens. D: default, P: parallel, E: on-edge.

Tensile specimens were designed and manufactured in accordance with ASTM D638-14 standard using sample type IV. In order to investigate the effect of print direction on the properties of the material, three different variations were considered as shown in Figure 1 and described here:

**Default parameters:**  Printed flat on the bed with two wall (outer) layers, resulting in a ±45° infill in the gauge section. Labelled **‘D’**.
**Parallel:**  Printed flat on the bed with 8 wall layers. This results in a parallel infill in the gauge section. Labelled **‘P’**.
**On-edge:**  Printed on its edge resulting in a print direction parallel to the loading direction for the whole sample. Labelled **‘E’**.

Increasing the number of wall layers for the parallel (P) specimens resulted in a discontinuity in the print path near the neck of the specimen. This was unavoidable due to limitations within the slicing; however, care was taken to ensure this discontinuity was as far from the gauge section as possible. To ensure printability, the on-edge (E) samples required a supporting structure under the raised gauge section. The supporting structure was automatically generated by *Eiger* and printed in the same material as the specimen. The support was removed after printing, leaving the underside of the specimen, particularly in the neck region, with some defects.

**Figure 2:**
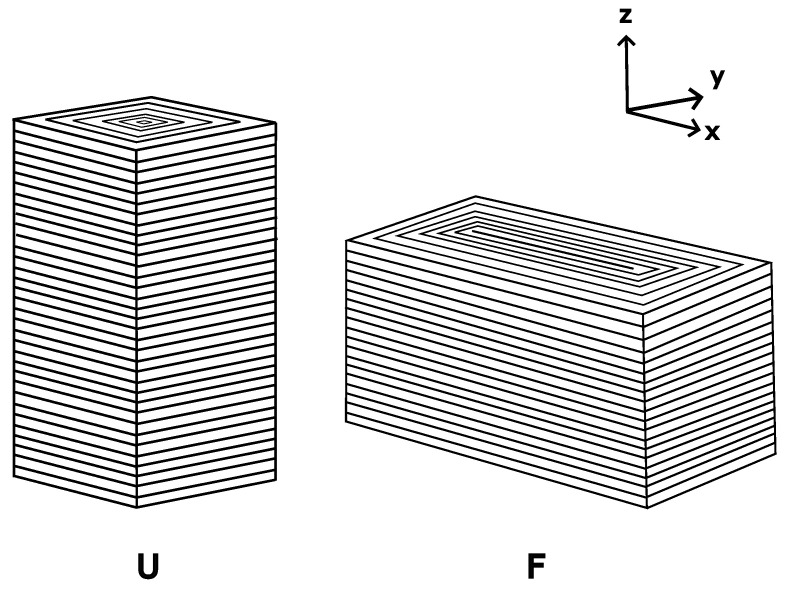
Infill of compression specimens. U: upright, F: flat.

Prism-shaped compression specimens were designed and manufactured in accordance with the ASTM D695-15 standard. The samples were printed in two orientations as shown in Figure 2 and herein described:

**Upright:**  Printed standing up on the bed with increased wall layers. This results in print layers being perpendicular to the loading direction. Labelled **‘U’**.
**Flat:**  Printed flat on the bed with increased wall layers. This results in the print layers being parallel to the loading direction. Labelled **‘F’**.

### 2.2. Testing

Testing was carried out over a range of strain rates using a universal Zwick Z100 Testing Machine. A charge-coupled device (CCD) camera was used to record the deformation. LA Vision Davis 8.4.0 DIC system was used to post-process the captured images and obtain strain fields on the specimens.

For tensile testing, the samples were placed in pincer grips spaced 65 mm apart and a 10 kN load cell was used to measure the force applied during the test. A minimum of four samples of each print configuration were tested to failure at each speed. The ASTM D638-14 standard specifies three crosshead speeds for tensile testing: 5, 50 and 500 mm/min. The maximum crosshead speed on the Zwick Z100 is 300 mm/min, and as such this was used in place of the 500 mm/min testing speed. The compressive samples were placed between two compression platens and compressed by 60% of their original height. The force was measured using a 100 kN load cell. To allow for the strain rate dependence to be investigated, three compression speeds were used. The crosshead speeds and corresponding nominal strain rates are shown in Table 1.

## 3. Results and Discussion

### 3.1. Tensile

#### 3.1.1. Onyx

**Figure 3:**
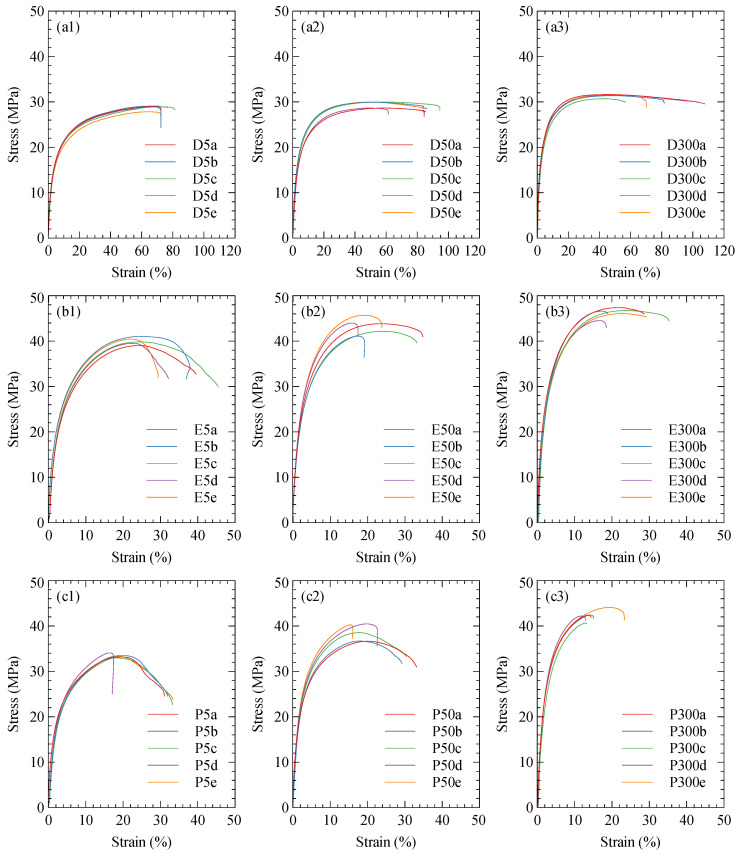
Onyx tensile stress–strain curves. The infill geometry is denoted by (**a**) default, (**b**) on-edge, and (**c**) parallel. Tensile speed denoted by (**1**) 5 mm/min, (**2**) 50 mm/min, and (**3**) 300 mm/min.

Stress–strain curves for the tensile Onyx specimens are shown in Figure 3. These curves demonstrate the strong dependence the tensile properties of Onyx have on the infill orientation.

**Figure 4:**
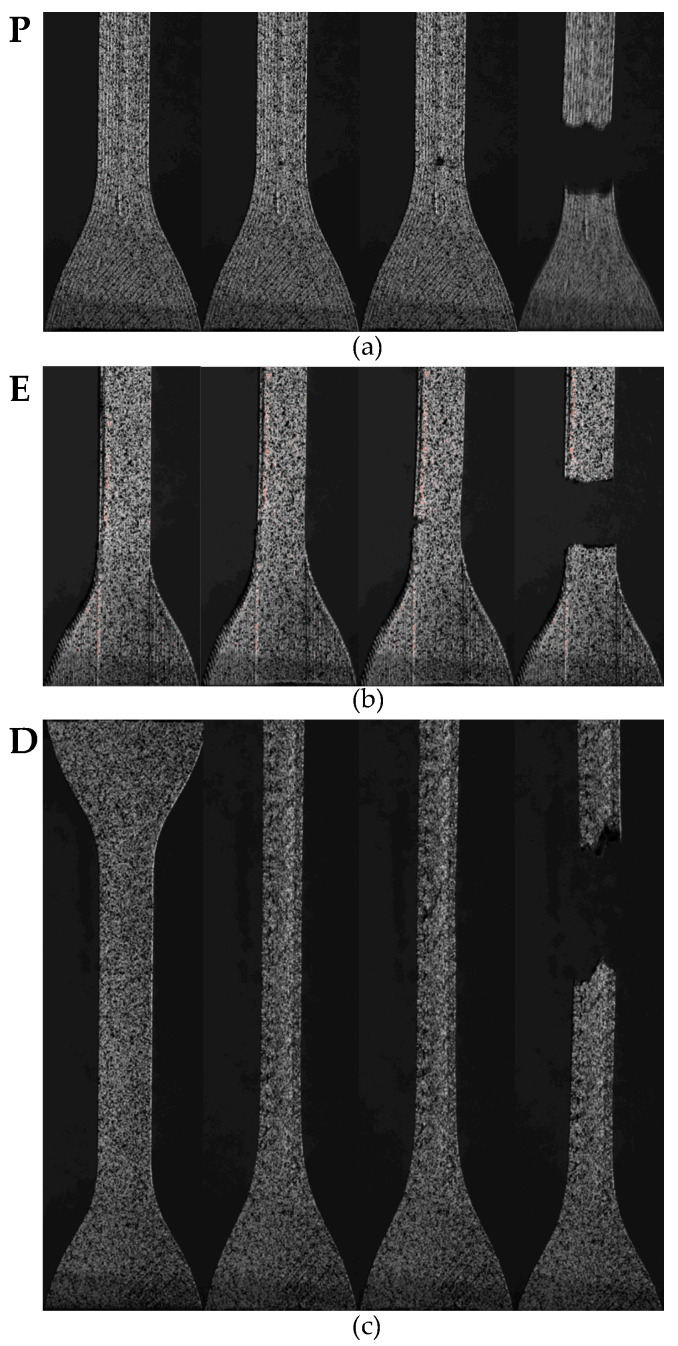
Failure mechanisms of tensile specimens showing (**a**) initiation and development of a void caused by a print defect in the parallel specimens; (**b**) initiation and development of a crack caused by a print defect in the on-edge specimens; and (**c**) failure in the gauge section of a standard specimen.

The on-edge and parallel specimens consistently failed sooner than the default specimens. Analysis of the images captured by the DIC system showed that these specimens failed due to defects in the printing. Figure 4a shows the initiation and development of a void at the discontinuity in the parallel specimens. This defect was caused by the change in print direction as mentioned in Section 2.1 and shown in Figure 1. This led to rupture and early failure of the sample. Similarly, failure was initiated at the neck region of the on-edge specimens, where the layers caused defects in the form of small steps in the curvature of the specimen (Figure 4b).

**Figure 5:**
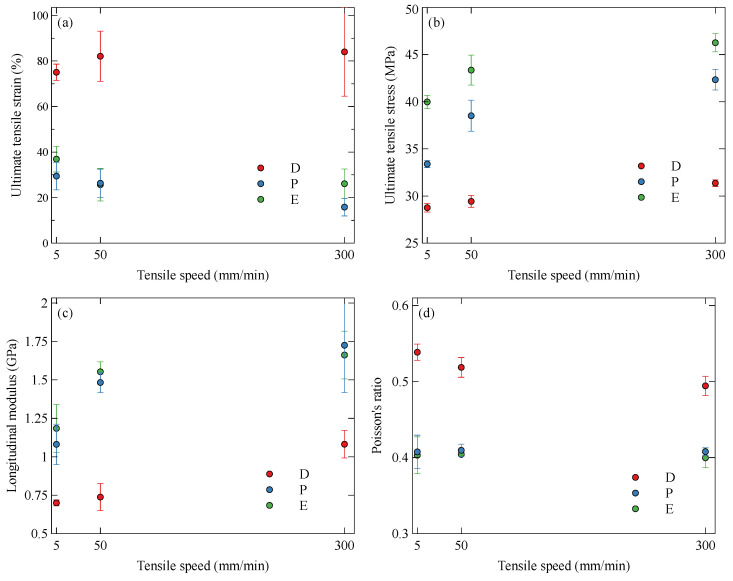
Variation of tensile properties of Onyx with strain rate and orientation: (**a**) ultimate tensile strain, (**b**) ultimate tensile stress, (**c**) longitudinal modulus, and (**d**) Poisson’s ratio for default (D), parallel (P) and on-edge (E) specimens.

The default specimens showed the longest elongation, up to around 100%, and these specimens tended to fail in the gauge section as in Figure 4c. Elongation to failure is shown more clearly in Figure 5a, where it is shown that the failure strain of the default specimens far exceeds that of the parallel and on-edge specimens. This occurs because the failure mechanisms of the parallel and on-edge specimens are governed by the geometrical defects, not the material, as seen in the default specimens. Across the range of tests, no correlation between failure strain and strain rate could be drawn.

The ultimate tensile stress (UTS) is seen to be influenced by both the strain rate and the infill orientation. Figure 5b shows a positive correlation between strain rate and UTS. This has been observed in previous studies [34,35,36] and is likely a result of a rate-dependent behaviour of the interfacial matrix–fibre adhesion, as suggested in [37]. The parallel and on-edge specimens demonstrate a higher UTS compared to the default. This is a result of fibre alignment during the extrusion process.

The initial modulus of the samples (Figure 5c) follows a similar trend to the UTS and is seen to increase with the strain rate. The on-edge and parallel specimens have a comparable modulus, and this can be attributed to the fact that both have a similar infill, whereby the short carbon fibres are oriented along the loading direction. The default specimens have a significantly lower modulus due to the off-axis infill.

The use of DIC allowed for the accurate determination of the Poisson’s ratio, which is plotted in Figure 5d. The Poisson’s ratio was calculated in the initial linear-elastic region of the tensile test by taking the ratio of the average lateral strain to longitudinal strain, both of which were given by the DIC analysis. The default Onyx samples, with a ±45° infill, displayed a significantly higher Poisson’s ratio than the parallel and on-edge samples. This is likely a result of a slight rotation of the infill to align with the loading direction during tension. Increasing the strain rate reduces the time allowed for this effect to take place, and the Poisson’s ratio is seen to decrease. The parallel and on-edge specimens both showed a comparable Poisson’s ratio, and this was consistent across the range of strain rates. This is due to the load acting parallel to the infill direction, which is the same for both specimens.

#### 3.1.2. Nylon

**Figure 6:**
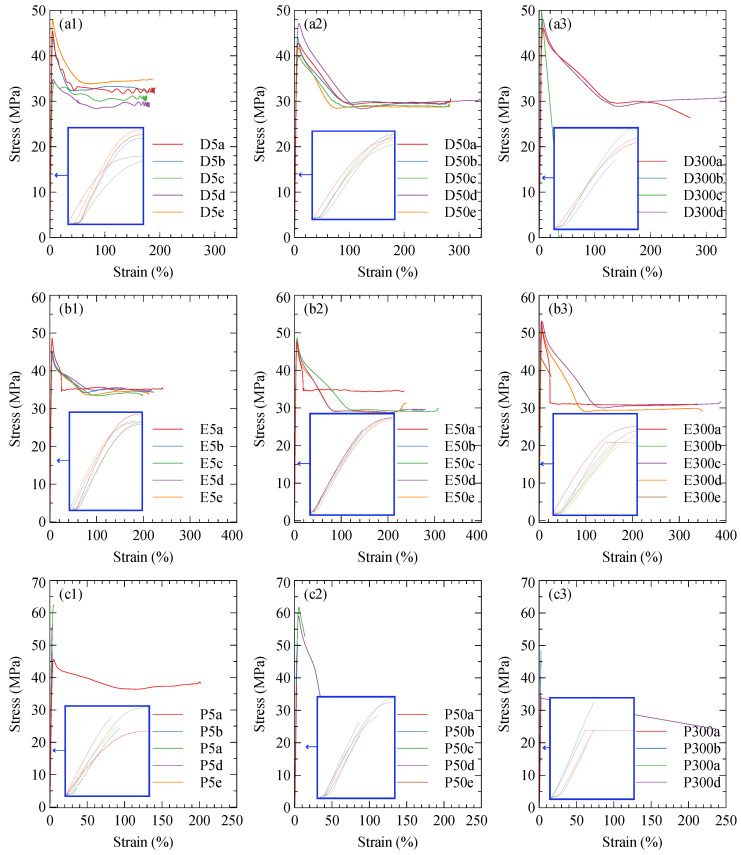
Nylon tensile stress–strain curves with first 5% strain inlaid. Infill geometry is denoted by (**a**) default, (**b**) on-edge, and (**c**) parallel. Tensile speeds are denoted by (**1**) 5 mm/min, (**2**) 50 mm/min, and (**3**) 300 mm/min.

**Figure 7:**
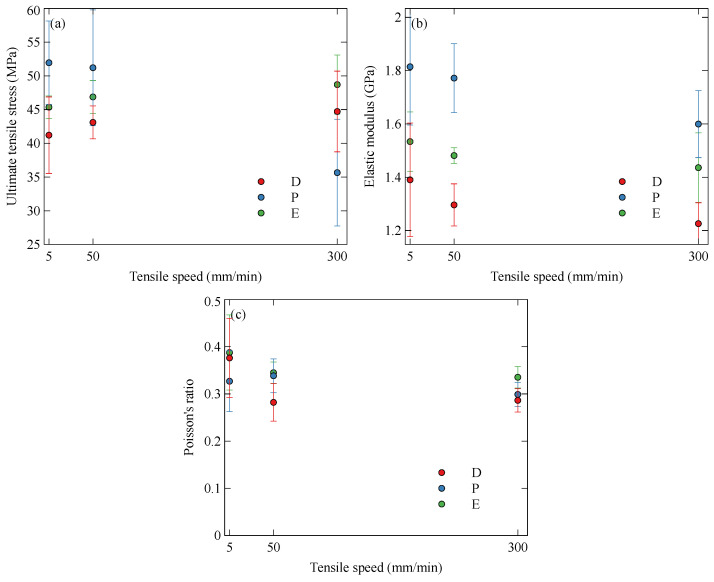
Variation of tensile properties of nylon with strain rate and orientation: (**a**) ultimate tensile stress, (**b**) longitudinal modulus, and (**c**) Poisson’s ratio.

Stress–strain curves for the nylon specimens are shown in Figure 6. Some samples did not undergo fast fracture, but instead a long plastic deformation with an increase in the elongation to failure with the strain rate. The UTS, plotted in Figure 7a, of the default and on-edge specimens show no significant variation with the strain rate. The parallel specimens failed primarily by fast fracture, resulting in a reduction in UTS with the strain rate. This is a result of the samples having less time to distribute the load away from the defect, thus causing a build-up of stress and a more rapid brittle fracture [38]. The high variance in the tests and the prevalence of fast fractures in the parallel specimens suggest that nylon is sensitive to defects in the printed samples.

It is shown in Figure 7b that the modulus of the samples may reduce slightly with the strain rate. The softening at increased strain rates could be a consequence of the pure nylon specimens having a lower thermal conductivity than the short carbon fibre-reinforced specimens in Section 3.1.1. This could result in the work performed during tension causing an increased temperature within the samples and increased softening.

The lack of fibre reinforcement in the nylon specimens leads to a lower initial Poisson’s ratio and lower dependence on the infill orientation due to the reduced anisotropy compared to the Onyx specimens. This is demonstrated in Figure 7c. Key tensile properties are tabulated in Table 2.

### 3.2. Compression

#### 3.2.1. Onyx

**Figure 8:**
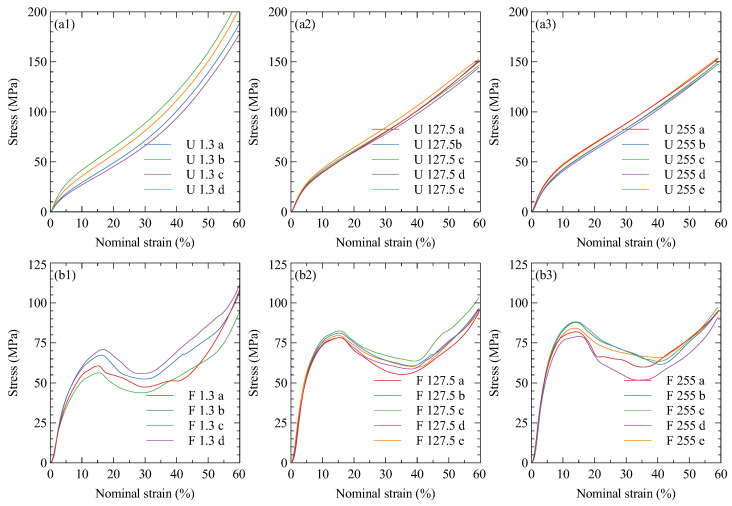
Onyx compressive stress–strain curves. Infill geometry is denoted by (**a**) upright, and (**b**) flat. Compressive speed denoted by (**1**) 1.3 mm/min, (**2**) 127.5 mm/min, and (**3**) 255 mm/min.

**Figure 9:**
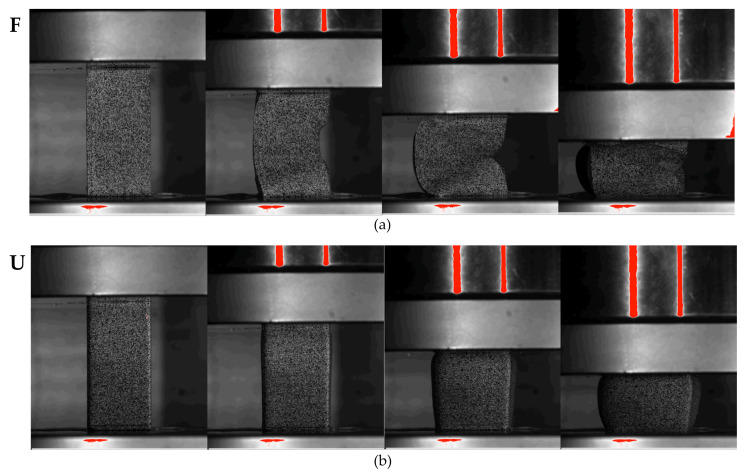
Compression behaviour of Onyx specimens showing (**a**) buckling behaviour of flat specimens; and (**b**) bulging behaviour of upright specimens.

**Figure 10:**
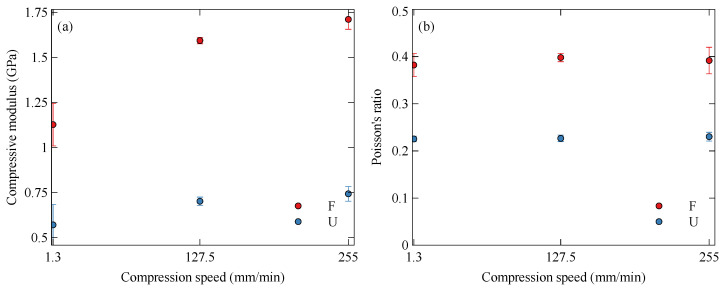
Variation of compressive properties of Onyx with strain rate and orientation: (**a**) compressive modulus and (**b**) Poisson’s ratio for flat (F) and upright (U) specimens.

The compressive behaviour of the Onyx samples was expected to be dependent on both the orientation of the infill and the applied strain rate; this is demonstrated well in Figure 8. A key difference is the buckling of the flat sample, which initiates at approximately 15% compression, as seen in Figure 9. During compression, the infill of the flat specimen is oriented parallel to the loading direction. Due to the fibre alignment during the printing process, the stiffness is therefore increased compared to the upright specimen, whose infill is perpendicular to the loading direction. This increased stiffness can be observed in Figure 10a, which compares the compressive modulus of the two orientations at a range of strain rates.

The use of DIC allowed the Poisson’s ratio of the compressive samples to be determined. This was calculated for the initial linear portion of the compression. The Poisson’s ratio showed no significant variation with strain rate, as can be seen in Figure 10b. The flat specimen, with the infill parallel to the loading direction, displays a significantly higher Poisson’s ratio than the upright printed specimen. This is due to the fibres in the upright specimen resisting the development of lateral strains. The numerical data is tabulated in Table 3.

#### 3.2.2. Nylon

**Figure 11:**
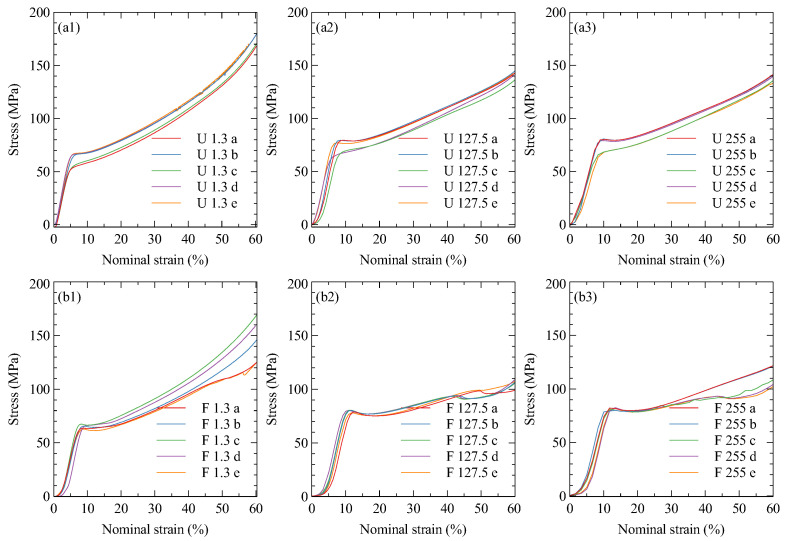
Nylon compressive stress–strain curves. Infill geometry denoted by (**a**) upright and (**b**) flat. Compressive speed is denoted by (**1**) 1.3 mm/min, (**2**) 127.5 mm/min, and (**3**) 255 mm/min.

**Figure 12:**
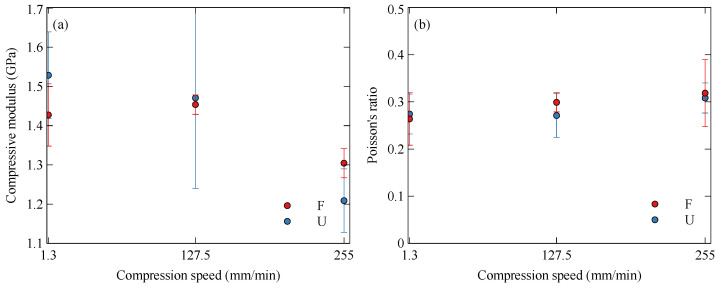
Variation of compressive properties of nylon with strain rate and orientation: (**a**) compressive modulus and (**b**) Poisson’s ratio.

The nylon samples all performed similarly under compression, as shown in Figure 11. The initial compressive modulus, Figure 12a, and the Poisson’s ratio, Figure 12b, show no significant dependence of the compressive properties on print orientation. The strain rate appears to have a subtle influence on the compressive properties, with a slight softening at higher strain rates along with an increase in Poisson’s ratio. Table 3 summarises these results.

## 4. Conclusions

In this paper, the tensile and compressive behaviour of additively manufactured reinforced and non-reinforced nylon was investigated. Short carbon fibre reinforcement, strain rate and infill orientation were all shown to affect the mechanical properties of the printed parts. Understanding the influence of these parameters will greatly help in the design for the manufacture of 3D-printed composite parts. The introduction of short carbon fibre reinforcement vastly improved the repeatability of the mechanical tests. The Onyx samples consistently had less test-to-test variation than the nylon samples whose properties and failures were far more sensitive to any defects or variations within the printing process.

The infill orientation affected the tensile properties for both the Onyx and nylon, with the parallel and on-edge specimens producing higher ultimate tensile stress (up to 47% and 37% higher respectively) and elastic modulus (up to 100% and 37% higher respectively). This was more pronounced in the Onyx samples due to the alignment of the fibres during the printing process. The Onyx samples had a higher tensile Poisson’s ratio than nylon with a strong dependence on infill orientation. A 34% increase in Poisson’s ratio was measured for the default Onyx specimens compared to the other orientations.

In compression, nylon showed no significant difference in compressive modulus for the two orientations compared. The Onyx specimens showed a 130% increase in compressive modulus of the flat specimens over the upright specimens. This is consistent with the expectation of a degree of reinforcement alignment with the load direction. In doing so, the sample was also more susceptible to local instability arising from the direct axial loading of the aligned reinforcement. The impact of the carbon fibre reinforcement was further emphasised when looking at the compressive Poisson’s ratio. The nylon specimens had an approximately consistent Poisson’s ratio, whereas the laterally oriented fibres in the upright Onyx specimens reduced the Poisson’s ratio by up to 43% compared to the flat alternative. Finally, the tensile and compressive moduli of Onyx showed a significant positive correlation with strain rate, with up to 60% and 50% increases, respectively, whereas the tensile and compressive moduli of Nylon exhibited a reduction of up to 10% and 20%. To understand this better, it would be beneficial in the future to measure the effect of strain rate on the temperature rise and the potential softening of the samples during tension.

It was demonstrated that the mechanical properties of 3D-printed reinforced and non-reinforced nylon are affected by the strain rate and print orientation. This is more pronounced in short fibre-reinforced nylon, where significant differences were observed. This is the first investigation of the parameters affecting the Poisson’s ratio of short carbon fibre-reinforced nylon, which will prove valuable for numerical modelling. Given the increase in additively manufactured fibre-reinforced polymers, it is clear that care needs to be taken both when characterising the mechanical properties and choosing the optimal design parameters.

## Figures and Tables

**Table 1:** Test speeds.

**Test Type**	**Crosshead Speed (mm/min)**	**Nominal Strain Rate (1/min)**
	5	0.15
Tensile	50	1.5
	300	9
	1.3	0.05
Compressive	127.5	5
	255	10

**Table 2:** Tensile properties of Nylon and Onyx: elastic modulus (E), Poisson’s ratio (ν) and ultimate tensile stress (UTS).

Material	Orientation	Test Speed (mm/min)	E (GPa)	ν	UTS (MPa)
Nylon	Default	5	1.39 ± 0.21	0.37 ± 0.08	41.21 ± 5.67
		50	1.30 ± 0.08	0.28 ± 0.04	43.11 ± 2.44
		300	1.23 ±0.08	0.29 ± 0.02	44.72 ± 5.98
	Parallel	5	1.81 ± 0.22	0.33 ± 0.06	51.95 ± 6.18
		50	1.77 ± 0.13	0.34 ± 0.04	51.21 ± 8.59
		300	1.56 ± 0.13	0.30 ± 0.03	35.65 ± 7.91
	On-edge	5	1.53 ± 0.11	0.39 ± 0.08	45.35 ± 1.69
		50	1.48 ± 0.03	0.34 ± 0.02	46.88 ± 2.44
		300	1.44 ± 0.13	0.34 ± 0.02	48.71 ± 4.39
Onyx	Default	5	0.70 ± 0.02	0.54 ± 0.01	28.75 ± 0.46
		50	0.74 ± 0.09	0.52 ± 0.01	29.42 ± 0.64
		300	1.08 ± 0.09	0.49 ± 0.01	31.36 ± 0.35
	Parallel	5	1.08 ± 0.13	0.40 ± 0.02	33.39 ± 0.37
		50	1.48 ± 0.07	0.40 ± 0.00	38.51 ± 1.65
		300	1.73 ± 0.31	0.40 ± 0.01	42.34 ± 1.10
	On-edge	5	1.18 ± 0.16	0.41 ± 0.02	39.98 ± 0.71
		50	1.55 ± 0.07	0.41 ± 0.01	43.35 ± 1.59
		300	1.66 ± 0.16	0.41 ± 0.01	46.26 ± 0.97

**Table 3:** Compressive properties of nylon and Onyx: compressive modulus (E) and Poisson’s ratio (ν).

Material	Orientation	Test Speed (mm/min)	E (GPa)	ν
Nylon	Flat	1.3	1.43 ± 0.08	0.26 ± 0.06
		127.5	1.45 ± 0.02	0.30 ± 0.02
		255	1.30 ± 0.04	0.32 ± 0.07
	Upright	1.3	1.53 ± 0.11	0.27 ± 0.04
		127.5	1.47 ± 0.23	0.27 ± 0.05
		255	1.21 ± 0.08	0.31 ± 0.03
Onyx	Flat	1.3	1.13 ± 0.12	0.38 ± 0.02
		127.5	1.59 ± 0.02	0.40 ± 0.01
		255	1.71 ± 0.05	0.39 ± 0.03
	Upright	1.3	0.57 ± 0.11	0.22 ± 0.01
		127.5	0.70 ± 0.02	0.22 ± 0.01
		255	0.74 ± 0.04	0.23 ± 0.01

## Data Availability

The data presented in this study are available on request from the corresponding author.

## Abbreviations

The following abbreviations are used in this manuscript:

AM	Additive manufacturing
SLA	Stereolithography
SLS	Selective laser sintering
FFF	Fused filament fabrication
DIC	Digital image correlation
CCD	Charge-coupled device
UTS	Ultimate tensile stress
